# IL-7 During Antigenic Stimulation Using Allogeneic Dendritic Cells Promotes Expansion of CD45RA^-^CD62L^+^CD4^+^ Invariant NKT Cells With Th-2 Biased Cytokine Production Profile

**DOI:** 10.3389/fimmu.2020.567406

**Published:** 2020-11-27

**Authors:** Abel Trujillo-Ocampo, Hyun-Woo Cho, Michael Clowers, Sumedha Pareek, Wilfredo Ruiz-Vazquez, Sung-Eun Lee, Jin S. Im

**Affiliations:** ^1^ Department of Stem Cell Transplantation and Cellular Therapy, University of Texas MD Anderson Cancer Center, Houston, TX, United States; ^2^ Department of Hematology, Seoul St. Mary’s Hospital, College of Medicine, The Catholic University of Korea, Seoul, South Korea

**Keywords:** *ex vivo* expansion, human iNKT cells, Th2 polarization of expanded iNKT cells, IL-2, IL-7, IL-15, αGalCer, CD62L^+^ iNKT cells

## Abstract

Invariant natural killer T (iNKT) cells are innate-like T lymphocytes cells that recognize glycolipid antigens associated with CD1d, non-classical antigen presenting proteins. They can drive either pro-inflammatory (Th-1) or anti-inflammatory (Th-2) immune microenvironment through the production of both Th-1 and Th-2 type cytokines upon activation, thus play a vital role in cancer, infection, and autoimmune diseases. Adoptive cell therapy using *ex vivo* expanded iNKT cells is a promising approach to enhance anti-tumor immunity or immunosuppression. However, overcoming phenotypic and functional heterogeneity and promoting *in vivo* persistency of iNKT cells remains to be a challenge. Here, we compared various methods for *ex vivo* expansion of human iNKT cells and assessed the quality of expansion, phenotype, and cytokine production profile of expanded iNKT cells. While a direct stimulation of iNKT cells in peripheral blood mononuclear cells with agonist glycolipid led to the expansion of iNKT cells in varying degrees, stimulation of enriched iNKT cells by irradiated autologous peripheral blood mononuclear cells or allogeneic dendritic cells resulted in consistent expansion of highly pure iNKT cells. Interestingly, the mode of antigenic stimulation influenced the dominant subtype of expanded iNKT cells. Further, we evaluated whether additional IL-7 or IL-15 during antigenic stimulation with allogeneic dendritic cells can improve the phenotypic heterogeneity and modify cytokine production profile of iNKT cells expanded from 18 consecutive donors. The presence of IL-7 or IL-15 during antigenic stimulation did not affect the fold of expansion or purity of expanded iNKT cells. However, IL-7, but not IL-15, led to a better expansion of CD4^+^ iNKT cells, enhanced Th-2 type cytokine production of CD4^+^ iNKT cells, and maintained the expansion of central memory (CD45RA^-^CD62L^+^) CD4^+^ iNKT cells. Our results suggest the addition of IL-7 during antigenic stimulation with allogeneic dendritic cells can promote the expansion of CD62L^+^Th-2^+^CD4^+^ human iNKT cells that can be used as novel immunotherapeutic to control excessive inflammation to treat various autoimmune diseases.

## Introduction

Invariant natural killer T (iNKT) cells are an innate lineage of T-cells that express a semi-invariant T-cell receptor (TCR) specific for glycolipid antigens presented by CD1d (1). The iNKT cells can influence adaptive immune responses through the production of a varying degree of both Th-1 and Th-2 type cytokines upon activation (2), thus play a critical role in various pathological conditions that occur during malignancy, infections, and autoimmune processes such as colitis, lupus, diabetes, and atherosclerosis (3–7). The exact molecular and cellular mechanisms of how iNKT cells regulate autoimmunity is yet to be elucidated, however, Th-2 type cytokines produced by iNKT cells are thought to promote immunosuppressive immune-microenvironment as well the differentiation of Th-2 T-cells while inhibiting the development of Th-1 T-cells (8).

Human iNKT cells are phenotypically and functionally diverse. The two main subsets, CD4^+^ and CD4^-^ iNKT cells, differ in their expression of Th-1 vs Th-2 type cytokines, effector molecules, and homing receptors (9–11). For example, CD4^-^ iNKT cells express a higher level of various natural killer receptors such as CD56, CD161, and NKG2D, and show greater cytotoxic activity than CD4^+^ iNKT cells. In contrast, CD4^+^ iNKT cells are better producers of Th-2 type cytokines such as IL-4 and IL-13 than CD4^-^ iNKT cells while producing similar levels of Th-1 type cytokines. These findings suggest that CD4^-^ iNKT cells may function as better effectors, whereas CD4^+^ iNKT cells may serve as better immunoregulators to control the immune microenvironment. As expanded human iNKT cells can be used as adoptive cell therapy to modulate adaptive immune cells to enhance anti-tumor immunity or immune-regulation (12, 13), it may be critical to obtaining a homogeneous population of iNKT cells that is optimized for either effector function (Th-1 polarized CD4^-^ iNKT cells) or regulatory function (Th-2 polarized CD4^+^ iNKT cells) in high purity with a clinically meaningful number.

Exogenous cytokines can influence the function of conventional T cells. For example, IL-15 promotes anti-tumor activity by supporting the expansion of CD8^+^ T cells (14) and the long-term survival of CD8^+^ memory T cells (15). Similarly, IL-7 can prolong the survival of CD4^+^ effector/memory T cells (16) and strengthen anti-tumor activity of CD4^+^ T cells (17). Both IL-7 and IL-15 have shown to play a pivotal role in the homeostasis of murine iNKT cells and may facilitate the expansion of human iNKT cells from adult peripheral blood and umbilical cord blood (18, 19). However, whether these cytokines can affect the selective expansion of human iNKT subsets or their cytokine production profile remains unknown.

In this study, we evaluated various strategies to expand iNKT cells and investigated if additional homeostatic growth factors such as IL-7 or IL-15 during antigenic stimulation improve the selective expansion of CD4^+^ or CD4^-^ iNKT cells and influences Th-1 vs Th-2-type cytokine production of expanded iNKT cells.

## Materials and Methods

### Materials

This study was performed in accordance with the research protocol approved by The University of Texas M.D. Anderson Institutional Review Committee and Institutional Biosafety Committee. Informed written consent from all study subjects was waived as all buffy coats from adult donors were purchased through the MDACC Blood Bank. Next, human peripheral blood mononuclear cells (PBMCs) from healthy adult donors were prepared from buffy coats by performing density gradient centrifugation using Histopaque-1077 Hybri-Max (Sigma Life Science, cat. no. H8889). All cells were cultured in T cell media (TCM)- RPMI-1640 medium containing L-glutamine (Gibco, cat. no. 11875-093) supplemented with 10% heat-inactivated fetal calf serum (FCS) (Hyclone, cat. no. A-1115-L), 0.001 mg/ml gentamicin (Gibco, cat. no. 15710-015), 0.1 mM nonessential amino acids (Gibco, cat. no. 11140-050), essential amino acids (Gibco, cat. no. 11130-051), 10 mM HEPES buffer solution (Gibco, cat. no. 15630-080), and 5.5 µM 2-mercaptoethanol (2-ME) (Gibco, cat. no. 21985-023). Recombinant Human IL-2 (Peprotech, # AF200-02), GM-CSF (Tonbo Cat #21-8339-CM01), and IL-4 (Tonbo Cat #21-8044-CM01) were purchased from either Peprotech or Tonbo. Lyophilized alpha-galactosylceramide (αGalCer, KRN7000) was purchased from Avanti lipids (# 867000), and dissolved in Dimethyl sulfoxide (Sigma, cat. no. D2650) at a concentration (100 µM).

### Antibodies

The following antibodies were purchased from Biolegend or BD Biosicence. iNKTCR (6B11, PE or PE-Cy7), CD4 (RPA-T4, APC or BV786), CD8α (SK11, PerCP-Cy5.5), IFN-γ (B27, Alexa Fluor 700), TNF-α (MAB11, APC), IL-4 (8D4-8, PE-Cy7), IL-13- (JES10-5A2, PE), and CD3 (OKT3, FITC or Pacific Blue). The iNK-TCR microbeads (#130-094-842) were purchased from Miltenyi biotech.

### Preparation of Antigen Presenting Cells

To obtain monocyte-derived dendritic cells (DCs), 5 x 10^8^ PBMCs were plated in a 150 cm^2^ flask in T cell medium (TCM) and incubated in a humidified incubator at 37°C/5% CO_2_ for 1 h. Subsequently, non-adherent cells were removed by gently rocking and aspirating, and remaining adherent monolayer of cells were cultured for 5 days in TCM supplemented with 200 U/ml recombinant human granulocyte/macrophage colony-stimulating factor and 100 ng/ml recombinant human IL-4. Cells were detached from the flask with 0.05 mM EDTA in PBS and treated with 5,000 rads of gamma irradiation using Cesium-137 irradiator to prevent proliferation prior to the use as antigen presenting cells for iNKT cells.

### Direct Expansion of iNKT Cells From PBMCs

Ten million of PBMCs from 15 consecutive healthy donors were placed in a well of 6 or 12 well plate tissue culture plate and incubated in 4–8 ml TCM containing αGalCer (200 nM) and IL-2 (200 IU/ml) for 14 days at 37°C. Cell culture was replenished with fresh media containing IL-2 200 IU/ml every 2–3 days. At day 14, cells were harvested and subjected to flow cytometric analysis of iNKT cells.

### Expansion of Enriched iNKT Cells Using α GalCer Pulsed Antigen Presenting Cells

The iNKT cells were enriched from up to 1x10^9^ PBMCs prepared from buffy coats using anti-iNKT MicroBeads (100µl of anti-iNKT Microbeads per 10^8^ total cells) in 400µl Magnetic-Activated Cell Sorting (MACS) buffer [PBS, pH 7.2, 0.5% human serum albumin-HSA (Sigma Life Science, cat. no. SRP6182)], and 2 mM EDTA (USB-Thermo Fisher, cat. no. 15694) according to manufacturer’s instruction. Subsequently the iNKT cells were isolated from the PBMCs using the MACS^®^ Cell Separation technique (Miltenyi Biotec) according to the company specified protocol using LS columns (Miltenyi Biotec, cat. no. 130-052-401). Subsequently, enriched iNKT cells were stimulated by irradiated antigen presenting cells (PBMCs, immature monocyte derived dendritic cells) pulsed with 200 nM αGalCer in presence of 200 IU/mL IL-2 for 10–14 days. For experiments to evaluate additive IL-7 and IL-15, 100 nM αGalCer was used to stimulate enriched iNKT cells using in irradiated dendritic cells in the presence of 10 ng/ml or 10 ng/ml IL-15 and 200 IU IL-2. Cell culture was replenished with fresh media containing corresponding growth factors only every 2–3 days.

### Flow Cytometry

Freshly isolated or expanded iNKT cells were subjected to multi-parameter flow cytometric analysis for the following markers: CD3, CD4, CD8α, CD25, CD45RA, CD62L, CD16, CD56, CD161, and invariant TCR α chain (6B11). Dead cells were excluded using fixable viable stain reagents (BD, cat. no. 564996). After staining for surface markers, cells were fixed and acquired using LSR Fortessa Cell Analyzer (BD Bioscience, Franklin Lakes, NJ). Flowjo version 10.3 (Tree Star, Ashland, OR) was used to analyze the expression of various surface antigens.

### Intracellular Cytokine Analysis

Expanded iNKT cells were stimulated in 96-well round-bottom cell culture plates (Corning) with phorbol 12-myristate 13-acetate (PMA, 30 ng/ml) and Ionomycin (1 ug/ml) for 3 h in the presence of GolgiStop (BD, cat. no. 554724) and GolgiPlug (BD, cat. no. 555029). Extracellular surface staining was performed first for surface markers such as CD3, CD4, invariant TCR α chain, and dead cells were stained with fixable viable stain reagents. These cells were fixed and permeabilized using Cytofix/Cytoperm (BD, cat. No. 51-2090KZ) and stained for various intracellular cytokines such as IFN-γ, TNFα, IL-4, and IL-13. All antibodies were purchased from BD Bioscience. Samples were acquired using LSR Fortessa Cell Analyzer, and FlowJo version 10.3 was used for analysis.

### The iNKT Cell Stimulation Assay

Fifty thousand iNKT cells were stimulated by 25,000 DCs pulsed with or without αGalCer (100 nM) in triplicates in 96-well plates for 48 h. Culture supernatants were assessed for the presence of IL-2, IL-4, IL-6, IL-10 TNFα, IFNγ, and IL-17A using Human Th1/Th2/Th17 Cytometric Bead Array (BD Bioscience, cat #560484) according to the manufacturer’s instructions.

For intracellular cytokine staining, iNKT cells after 48 h incubation with DCs with or without αGalCer were incubated for another 6 h in presence of protein transport inhibitor (containing 1 umol/L of monesin) and subjected to multi-parameter flow cytometric analysis for the following surface markers: CD3, CD4, iNK invariant TCRα chain (clone: 6B11). Dead cells were excluded using Fixable Viable Stain 620 (BD Bioscience). Subsequently, cells were fixed and permeabilized using BD Cytofix/Cytoperm (BD bioscience) and stained with the next cytokines: IL-4, IFNγ and TNFα. Samples were acquired using LSR Fortessa Cell Analyzer, and FlowJo version 10.3 was used for analysis.

### Statistical Analysis

Analyses were performed using GraphPad Prism version 8.00 for Windows GraphPad Software (La Jolla, California). Datasets were analyzed using a two-tailed non-parametric, paired t-test with confidence level: 95%, and *p* values for comparisons between groups/conditions were determined. A *p-*value less than 0.05 was considered statistically significant.

## Results

### Direct Expansion of iNKT Cells in Peripheral Blood Mononuclear Cells Failed to Achieve Consistent and Selective Expansion of iNKT Cells

Human peripheral blood mononuclear cells (PBMCs) prepared from ficoll density gradient have a mixture of various immune subsets consisting of not only circulating T cells, B cells, natural killer cells but also various antigen presenting cells such as monocytes and dendritic cells. Therefore, the direct addition of cognate antigen to this mixture of immune cells is often sufficient to expand pre-existing memory T cell populations. As iNKT cells are considered as innate memory T cells, it is feasible to expand iNKT cells from PBMCs by adding αGalCer and IL-2, the growth factor for T cells. Therefore, we evaluated whether direct stimulation can effectively and reliably expand iNKT cells in consistent quality, and assessed the purity and phenotype of expanded iNKT cells from PBMCs of 15 consecutive donors.

**Figure 1:**
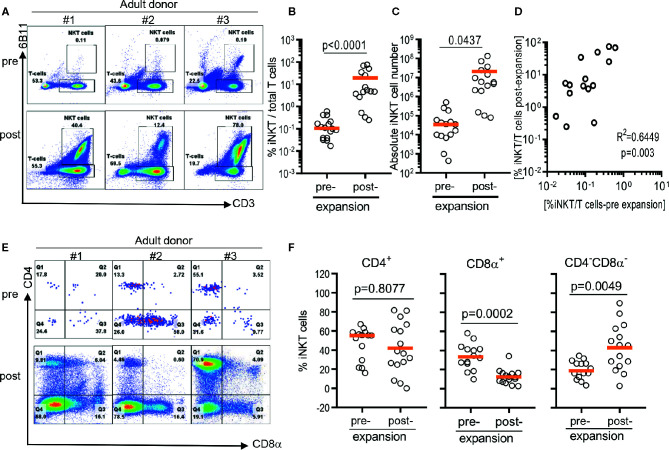
Direct antigenic stimulation of iNKT cells from peripheral blood mononuclear cells (PBMCs). **(A)** We performed flow cytometry analysis pre (day 0) and post (day 10) that iNKT cells gated on 6B11 and CD3. Fresh isolated iNKT cells from 3 healthy donors were stimulated with αGalCer pulsed PBMCs by use of 200 nM/ml αGalCer in the presence of 200 U/ml IL-2 for 10 days. **(B)** iNKT cells from 15 healthy donors were stimulated with αGalCer pulsed PBMCs by use of 200 nM/ml αGalCer in the presence of 200 U/ml IL-2 for 10 days. The percentage indicates the 6B11^+^ and CD3^+^ iNKT cells. **(C)** Absolute numbers in pre- and post- expansion from direct antigenic stimulation of iNKT cells from PBMCs. **(D)** Linear regression showing the correlation between the percentage of pre and post of iNKT cells in healthy donor samples. **(E, F)** Analysis of the phenotype of CD4^+^, CD8α^+^, and CD4^-^CD8^-^ iNKT cells at day 10. A paired *t* test was used to compare the differences between selected groups.

As previously reported (12, 20), iNKT cells were present in about a median 0.105% of circulating T cells with a large donor to donor variation raining from 0.0137% to 0.5930% (**Figures 1A, B**). After a single round of stimulation using αGalCer, the frequency of iNKT cells significantly increased to median 4.85% ranging from 0.25% to 75% (**Figure 1B**). The presence of higher frequencies in PBMCs was correlated to higher purity of iNKT cells post expansion (r^2^ = 0.6449, p = 0.003) (**Figure 1D**). Direct stimulation of iNKT cells in PBMCs achieved median 69.4 folds of expansion with a range of 1.9 to 448.9 fold but was not correlated to the presence of higher percentage of iNKT cells in either pre- or post- expansion culture (**Figure 1D**).

The iNKT cells are phenotypically heterogeneous, consisting of CD4^+^, CD8α^+‑^, or CD4^-^CD8a^-^ (DN) in varying degrees. Predominant phenotype of iNKT cells among 15 donors was CD4^+^ (median 55.2%, range 16.0% to 62.0%), followed by CD8α^+^ (median 33.2%, range 9.7% to 58.0%), and CD4^-^CD8a^-^ phenotype (median 18.6%, range 3.3% to 34.4%) (**Figures 1E, F**). However, *ex vivo* expansion of iNKT cells led to significant phenotypic alteration in expanded iNKT cells. While there was no significant change in percentage of CD4^+^ phenotype, CD8α^+^ iNKT cells has significantly decreased to median 11.9% (range: 2.5% to 31.5%) with reciprocal increase in CD4^-^CD8α^-^ (DN) phenotype to median 45.3% (range 2.7% to 86.6%) (**Figures 1E, F**).

In summary, the direct stimulation of iNKT cells in peripheral blood mononuclear cells led the expansion of iNKT cells in variable degrees and altered heterogeneity in phenotype.

### The Initial Enrichment of iNKT Cells and Subsequent Antigen Specific Stimulation Using Irradiated Antigen Presenting Cells Resulted in Effective Expansion of Highly Pure iNKT Cells

**Figure 2:**
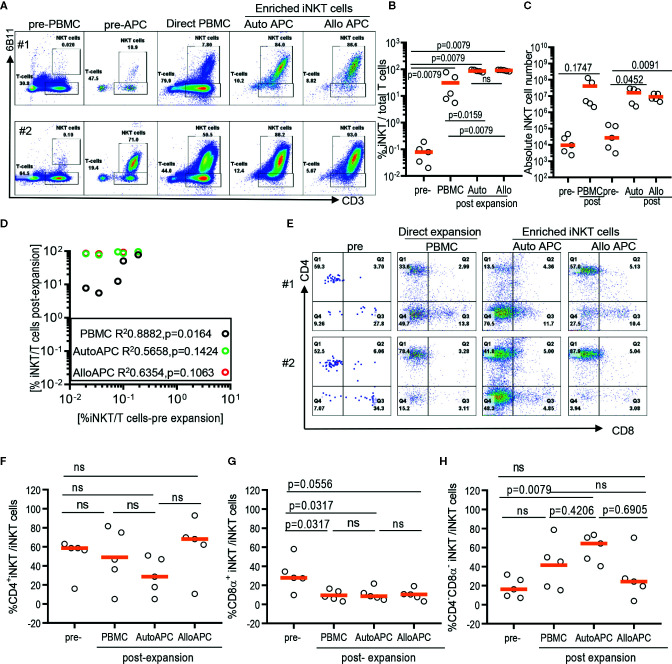
Expansion of enriched iNKT cells via antigenic stimulation using αGalCer pulsed irradiated autologous peripheral blood mononuclear cells (PBMCs) and allogeneic dendritic cell (DC). The iNKT cells were enriched from five healthy donors and co-cultured with irradiated autologous PBMCs or allogeneic DC in the presence of αGalCer and IL-2 for 10 days. As a control, αGalCer and IL-2 were directly added to PBMCs. **(A)** The representative flow cytometric analyses of iNKT cells pre and post expansion from two donors. **(B)** Percentage of iNKT cells from total T cells at each step from five donors. **(C)** Absolute numbers in pre- and post- expansion from direct antigenic stimulation of iNKT cells from PBMCs and autologous and allogenic DCs. **(D)** Linear regression showing the correlation between the percentage of pre and post of iNKT cells in healthy donor samples. **(E–H)** The percentage of CD4^+^, CD8α^+^ and CD4^-^CD8α^-^ iNKT cells pre and post expansion. A paired *t* test was used to compare the differences between selected groups.

Because iNKT cells are a rare population that comprises 0.001% up to 3% of circulating T cells with a large donor-to-donor variation, it may contribute to the difficulty in obtaining highly pure iNKT cells from the pool of peripheral blood immune cells *via* direct stimulation of iNKT cells in PBMCs (**Figures 2A, B**). Therefore, we evaluate whether the initial enrichment step of iNKT cells can overcome relative paucity and donor-to-donor variation of iNKT cells and yield the consistent expansion of iNKT cells in high quality. First, iNKT cells from PBMCs were enriched *via* magnetic activated cell sorting (MACS) technique with commercially available anti-iNK TCR microbeads, then co-cultured enriched iNKT cells with irradiated autologous PBMCs in the presence of αGalCer. The percentage of iNKT cells were significantly increased from average 0.1% (range 0.039% to 0.42%) to 35.7% (range 7.07 to 85.5%) after one-step enrichment, and subsequent stimulation of enriched iNKT cells using αGalCer pulsed autologous PBMCs substantially increased the purity of iNKT cells to average 96.60% (range: 86.7% to 99.3%) (**Figures 2A, B**). This is a significantly selective expansion of iNKT cells as the direct stimulation of iNKT cells in PBMCs from the same donors resulted in only an average 7.47% (range: 2.71% to 75.0%). Lastly, we did not observe any significant differences in fold of expansion or absolute number of iNKT cells between direct stimulation vs using autologous or allogeneic APC (**Figures 2C, D**). Our findings suggest that the initial enrichment of iNKT cells was an essential step to obtain highly pure iNKT cells after *ex vivo* expansion.

As iNKT cells are restricted to monomorphic CD1d antigen presenting molecule, allogeneic antigen presenting cells can be alternative antigen presenting cells to expand iNKT cells. Therefore, we explore whether allogeneic immature dendritic cells can be used instead of autologous PBMCs as monocyte-derived, allogeneic dendritic cells may offer effective stimulation of T cells over monocytes – the majority of antigen presenting cells present in PBMCs. Antigenic stimulation using allogeneic dendritic cells led to as efficient expansion of iNKT cells as stimulation using autologous APC, with average 97% (range: 90.5% to 99.5%) vs 96.6% (range: 86.7% to 99.3%) (**Figures 2B, D**), respectively. Our results support that allogeneic immature dendritic cells can be used interchangeably to autologous PBMCs for delivering antigenic stimulation to iNKT cells.

Lastly, we assessed whether different modes of antigenic stimulation could affect the composition of CD4^+^ vs CD4^-^ iNKT cells after *ex vivo* expansion (**Figures 2E–H**). Since CD4^+^ vs CD4^-^ iNKT cells display overlapping yet a distinct range of functional heterogeneity in terms of Th1 vs Th2 cytokine production profile and cytotoxicity (10, 12), it is important to obtain as homogenous population as possible in order to maximize anti-tumor immunity or anti-inflammatory properties of expanded iNKT cells as cell therapy product. Previously, we observed that the stimulation of polyclonal iNKT cells *via* αGalCer pulsed allogeneic dendritic cells preferentially expanded CD4^+^ iNKT cells with a reciprocal decrease in CD4^-^CD8α^-^ iNKT cells (12), and reported here that the direct stimulation of iNKT cells from PBMCs preferentially expanded CD4^-^CD8α^-^ iNKT cells (**Figure 1**). Head to head comparison of different antigenic stimulation revealed that stimulation with allogeneic dendritic cells led to a trend toward an increase in CD4^+^ iNKT cells with a significant decrease in CD8α^+^ or CD4^-^CD8α^-^ iNKT cells compared to direct stimulation of iNKT cells or antigenic stimulation by autologous PBMCs. On the contrary, αGalCer pulsed autologous PBMCs resulted in a significant expansion of CD4^-^CD8α^-^ iNKT cells with reciprocal reduction of CD4^+^ or CD8α^+^ iNKT cells after antigenic stimulation, compared to the direct stimulation of iNKT cells or αGalCer pulsed allogeneic dendritic cells. Our works demonstrated that the mode of antigenic stimulation for iNKT cells drove a differential expansion of iNKT subsets, and should be under careful consideration when to attempt developing cell therapy using expanded iNKT cells.

### IL-7 During Dendritic Cells Mediated Antigenic Stimulation and Augmented the Expansion of CD4^+^ and CD4^+^CD25^+^ iNKT Cells

As CD4^+^ iNKT cells produce a higher level of Th-2 type cytokines than CD4^-^ iNKT cells (10, 12), these highly enriched CD4^+^ human iNKT cells have the potential to be used as novel cell therapy to modulate immune microenvironment towards immune-suppression in several autoimmune processes such as graft versus host disease (12, 21). Previously, we showed that antigenic stimulation with allogeneic dendritic cells can help a preferential expansion of CD4^+^ iNKT cells while CD4^-^CD8α^-^ iNKT cells are predominant subset of iNKT cells expanded *via* αGalCer pulsed autologous PBMCs (**Figure 2**) (12). Here, we explored whether additional cytokines – IL-7 or IL-15, homeostatic cytokines for CD4^+^ vs CD4^-^ iNKT cells, can further facilitate the selective expansion of CD4^+^ iNKT cells.

**Figure 3:**
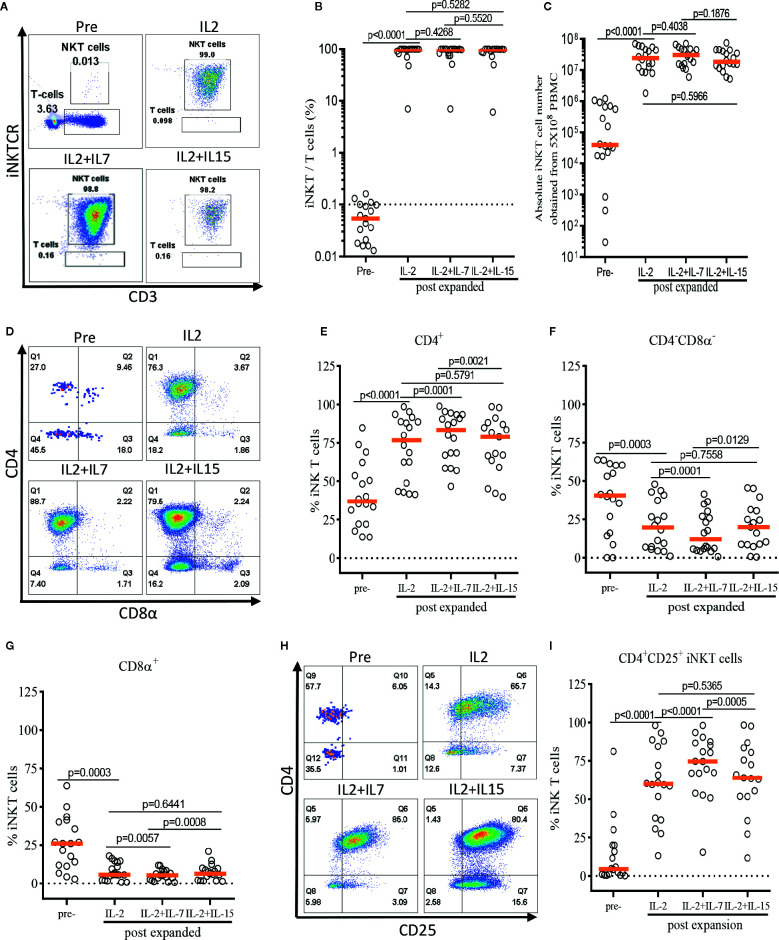
IL-7 stimulation preferentially expands human CD4^+^ and CD4^+^CD25^+^ iNKT cells. The iNKT cells were enriched from peripheral blood mononuclear cells (PBMCs) *via* anti-iNKT microbeads/MACS separation and subsequently co-cultured with allogeneic dendritic cells (DCs) in presence of αGalCer and growth factors (IL-2, IL-2+IL-7, or IL-2+IL-15) for 14 days. **(A–C)** Representative dot-plot flow cytometry analysis, purity, and absolute number of iNKT cells in pre- and post-expansion. **(D–G)** Representative flow cytometry analysis and the percentage of CD4^+^, CD8α^+^, and CD4^-^CD8α^-^ iNKT cells in pre- and post-expansion. **(H, I)** Representative flow cytometry analysis of CD4 and CD25 on iNKT cells in pre- and post-expansion. Horizontal orange lines show median; *p* values are shown above each group. Each symbol represents a value from a single donor.

First, we assessed the influence of IL-7 and IL-15 on the quality and quantity of the *ex vivo* expansion of human iNKT cells (**Figure 3**). The purity of iNKT cells increased from an average of 0.05% in peripheral blood to an average of 98.74%, 98.53%, and 98.49% after antigen-specific expansion in the presence of IL-2, IL-2+IL-7, and IL-2+IL-15, respectively (**Figure 3B**). An average of 3.18 x 10^5^ iNKT cells were obtained after MACS enrichment from 5 x 10^8^ PBMCs, and these cells underwent a robust antigenic expansion to yield an average of 2.97 x 10^7^, 3.24 x 10^7^, and 2.60 x 10^7^ iNKT cells in presence of IL-2, IL-2+IL-7, and IL-2+IL-15, respectively (**Figure 3C**). There were no significant differences in the purity as well as viability (**Supplementary Figure 1**) and quantity (or fold of expansion, **Figure 3C**) of expanded iNKT cells among the three culture conditions.

Next, we assessed the impact of IL-7 or IL-15 on the selective expansion of CD4^+^ or CD4^+^CD25^+^ iNKT cells. As expected, the percentage of CD4^+^ iNKT cells increased significantly after expansion in all groups as compared with freshly isolated iNKT cells, with a reciprocal reduction in percentages of CD4^-^CD8α^-^ and CD8α^+^ iNKT cells (**Figures 3D–G**). Interestingly, the addition of IL-7, but not IL-15, to the antigenic stimulation further aided the expansion of CD4^+^ iNKT cells (*p=*0.0001). Lastly, we investigated the induction of CD25 expression on the CD4^+^ iNKT cells, a marker suggestive of regulatory properties and activation (**Figures 3H–I**). We observed a significant expansion of the CD4^+^CD25^+^ iNKT population after antigenic stimulation using IL-2 alone compared with freshly isolated iNKT cells. Again, IL-7, but not IL-15, significantly augmented the expression of CD25 on CD4^+^ iNKT cells when added to co-culture (*p<*0.0001).

In summary, we demonstrated that addition of IL-7, but not IL-15, to a single antigenic stimulation, promoted the selective expansion of CD4^+^ and CD4^+^CD25^+^ iNKT cells while not affecting the quality and quantity of the *ex vivo* expansion of iNKT cells.

### IL-7, but Not IL-15, During Antigenic Stimulation Maintained CD62L^+^ Central Memory iNKT Cells

**Figure 4:**
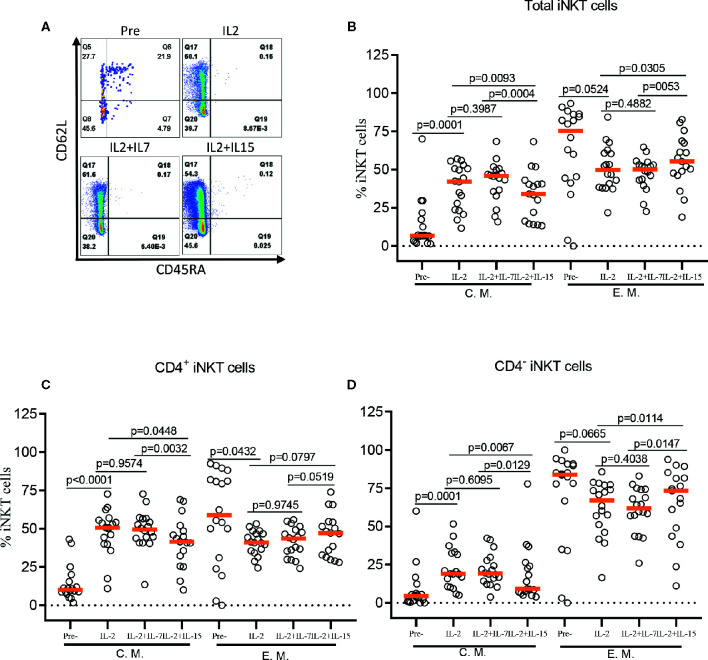
IL-7 but not IL-15 supports the expansion of CD62L^+^ central memory iNKT cells. **(A)** Representative flow cytometry analysis of CD62L and CD45RA expression on total iNKT cells before and after antigen specific expansion with IL-2, IL-2+IL-7, and IL-2+IL-15. **(B–D)** The percent of central memory (CM, CD45RA^-^CD62L^+^) and effector memory (EM, CD45RA^+^CD62L^-^) phenotype of total, CD4^+^, and CD4^-^ iNKT cells in pre- and post-expansion. Horizontal orange lines show median values; *p* values are shown above each group. Each symbol represents a value from a single donor.

The role of IL-7 and IL-15 in differentiation and maintenance of conventional memory T-cells (22, 23), as well as homeostasis of murine and human iNKT cells (24–26) has been well established. Here, we investigated the effect of IL-7 or IL-15 during *ex vivo* expansion on human iNKT cell differentiation into memory phenotypes such as naïve (CD45RA^+^CD62L^+^), central memory (CM, CD45RA^-^CD62L^+^), effector memory (EM, CD45RA^-^CD62L^-^), and effector (EFF, CD45RA^+^CD62L^-^) (**Figure 4**). This is particularly relevant because CD62L^+^ gene-modified iNKT cells can exert better anti-tumor activity likely due to prolonged persistence (19). Antigen-specific stimulation with IL-2 alone resulted in a significant expansion of CM iNKT cells compared with freshly isolated iNKT cells, with a reciprocal decrease in EM iNKT cells (12). While the addition of IL-7 during antigenic stimulation did not affect the expansion of CM iNKT cells, IL-15 led to a less effective expansion of CM iNKT cells, in total and CD4^+^ iNKT cells, compared with IL-2 (total iNKT cell, *p*=0.0093 & CD4^+^ iNKT cell, *p*=0.0448) alone or IL-2+IL-7 (total iNKT cell, *p*=0.0004 & CD4^+^ iNKT cell, *p*=0.0032) (**Figure 4**). Our results suggest that IL-7, but not IL-15, supported maintaining CD62L^+^ CM iNKT cells when supplemented during antigenic stimulation of freshly isolated human iNKT cells.

### IL-7 During Antigen Stimulation Promoted the Production of Th-2 Type Cytokines by Expanded CD4^+^ iNKT Cells

Conventional CD4^+^ T helper cells can differentiate into Th-1 or Th-2 subsets upon activation and produce sets of inflammatory (Th-1) or anti-inflammatory (Th-2) type cytokines (27). Differently from conventional T cells, iNKT cells can produce both Th-1 and Th-2 type cytokines within several hours of activation. Th-1 type responses of iNKT cells are critical for optimizing anti-tumor and microbial adaptive immunity (28–30), while Th-2 type responses of iNKT cells may play a role in preventing excessive inflammation in several autoimmune process such as diabetes, lupus, allergic reaction, and graft versus host disease (5, 7, 21, 31). Therefore, functional polarization during *ex vivo* expansion of iNKT cells may be important to maximize their inflammatory or anti-inflammatory properties for adoptive cell therapy.

Both CD4^+^ and CD4^-^ iNKT cells can produce Th-1 cytokines in similar levels, but CD4^+^ iNKT cells can produce Th-2 type cytokines in a larger amount than CD4^-^ iNKT cells. This intrinsic ability to produce Th-2 type cytokines can contribute to the functional polarization of iNKT cells, and this relative ratio of Th-1 vs Th-2 type cytokine production can be altered by several factors such as qualitative and quantitative differences in antigenic stimuli, growth factors, or extracellular cytokines (10, 11, 32, 33). In this study, we have already demonstrated that the presence of IL-7 during antigenic stimulation enhanced the selective expansion of CD4^+^ iNKT cells. Here, we examined how the addition of IL-7 or IL-15 during antigenic stimulation in *ex vivo* affected the cytokine production profile of expanded iNKT cells.

**Figure 5:**
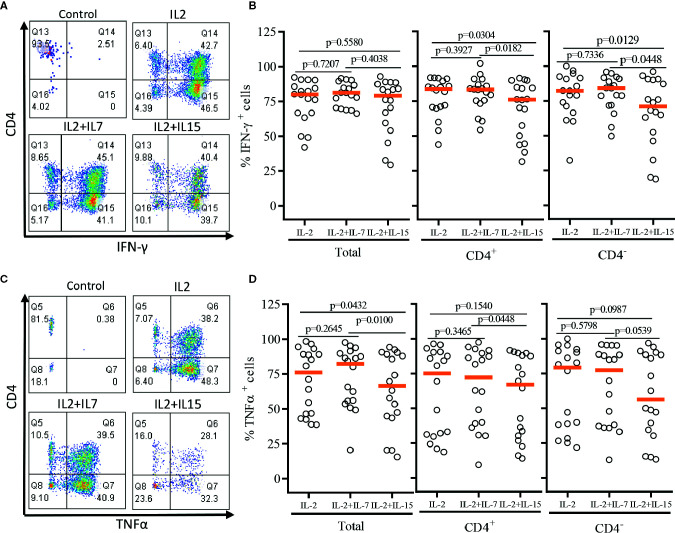
Th-1 type cytokine production profile of expanded human iNKT cells. The expanded iNKT cells were stimulated with PMA and Ionomycin and intracellular cytokine production profiles were assessed through multi-color flow cytometry. **(A)** Representative flow cytometric analysis of CD4 and IFNγ of iNKT cells in post-expansion. **(B)** Percent IFNγ^+^ total, CD4^+^, and CD4^-^ iNKT cells in post-expansion with IL-2, IL-2+IL-7, and IL-2+IL-15. **(C)** Representative flow cytometric analysis of CD4 and TNFα of iNKT cells in post-expansion. **(D)** Percent TNFα^+^ total, CD4^+^, and CD4^-^ iNKT cells in post-expansion with IL-2, IL-2+IL-7, and IL-2+IL-15. Horizontal lines represent median values; *p* values are shown above each group. Each symbol represents a value from a single donor.

**Figure 6:**
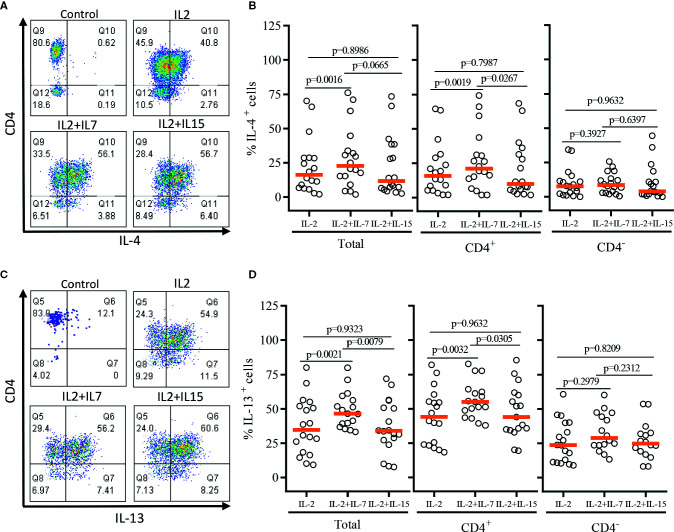
Th-2 type cytokine production profile of expanded human iNKT cells. The expanded iNKT cells were stimulated with PMA and Ionomycin, and intracellular cytokine production profiles were assessed through multi-color flow cytometry. **(A)** Representative flow cytometric analysis of CD4 and IL-4 expression of iNKT cells post-expansion. **(B)** Percent of IL-4^+^ total, CD4^+^, and CD4^-^ iNKT cells in post-expansion with IL-2, IL-2+IL-7, and IL-2+IL-15. **(C)** Representative flow cytometric analysis of CD4 and IL-13 expression of iNKT cells in post-expansion. **(D)** Percent IL-13^+^ total, CD4^+^ and CD4^-^ iNKT cells in post-expansion with IL-2, IL-2+IL-7, and IL-2+IL-15. Horizontal orange lines represent median values; *p* values are shown above group. Each symbol represents a value from a single donor.

After a single round of antigenic stimulation, we evaluated Th-1 and Th-2 cytokine production of expanded iNKT cells through intracellular cytokine analysis following activation by PMA/Ionomycin. CD4^+^ iNKT cells produced higher level of Th-2 type cytokines (IL-4 and IL-13) than their counterpart CD4^-^ iNKT cells, while producing similar levels of Th-1 type cytokines (IFNγ and TNFα) to CD4^-^ iNKT cells (**Figures 5** and **6**). The addition of IL-7 during antigenic stimulation did not affect the level of Th-1 type cytokine production of both CD4^+^ and CD4^-^ iNKT cells, but IL-15 led to a trend of decreasing Th-1 type cytokine production in CD4^-^ iNKT cells (**Figure 5**). On the other hand, the addition of IL-7 during antigenic stimulation significantly increased IL-4^+^ and IL-13^+^ iNKT cells in total (IL-4, *p*=0.0016 & IL-13, *p*=0.0021) and CD4^+^ (IL-4, *p*=0.0019 & IL-13, *p*=0.0032) subset. Lastly, IL-15 did not influence Th-2 type cytokine production of both CD4^+^ and CD4^-^ iNKT cells.

**Figure 7:**
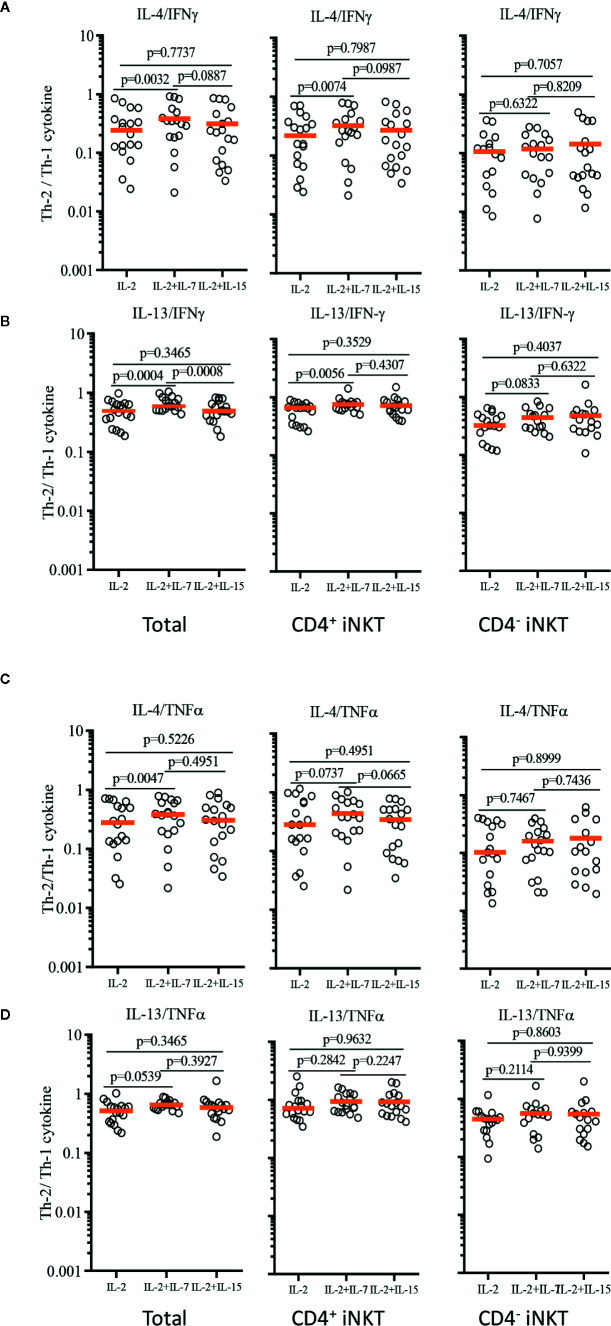
Ratios of Th-2 to Th-1 cytokine production profile of expanded human iNKT cells. Ratios of Th-2 cytokine (IL-4 and IL-13)^+^ iNKT cells to Th-1 cytokine^+^ iNKT cells were calculated for IL-4/IFNγ **(A)**, IL-13/IFNγ **(B)**, IL-4/TNFα **(C)**, IL-13/TNFα **(D)** from iNKT cells after antigenic stimulation with IL-2, IL-2+IL-7, and IL-2+IL-15. Horizontal orange lines represent median values; *p* values are shown in the above each group. Each symbol represents a value from a single donor.

In order to demonstrate the relative Th-1 vs Th-2 polarization of CD4^+^ iNKT cells vs paired CD4^-^ iNKT cells, we calculated the ratio of Th-2^+^ to Th-1^+^ cytokine-producing iNKT cells to total, CD4^+^, and CD4^-^ iNKT cells (**Figure 7**). The analysis confirmed that the addition of IL-7 to co-culture significantly increased the ratio of Th-2 to Th-1 cytokines (IL-4/IFNγ and IL-13/IFNγ) on total (IL-4/IFNγ, *p*=0.0032 & IL-13/IFNγ, *p*=0.0004) and CD4^+^ (IL-4/IFNγ, *p*=0.0074 & IL-13/IFNγ, *p*=0.0056) iNKT cells compared to those co-cultured with IL-2 alone. This synergistic increase was not present with the addition of IL-15. There was a trend of higher ratio of IL-4^+^ or IL-13^+^ to TNFα^+^ iNKT cells expanded with IL-2 + IL-7, but this was not statistically significant.

**Figure 8:**
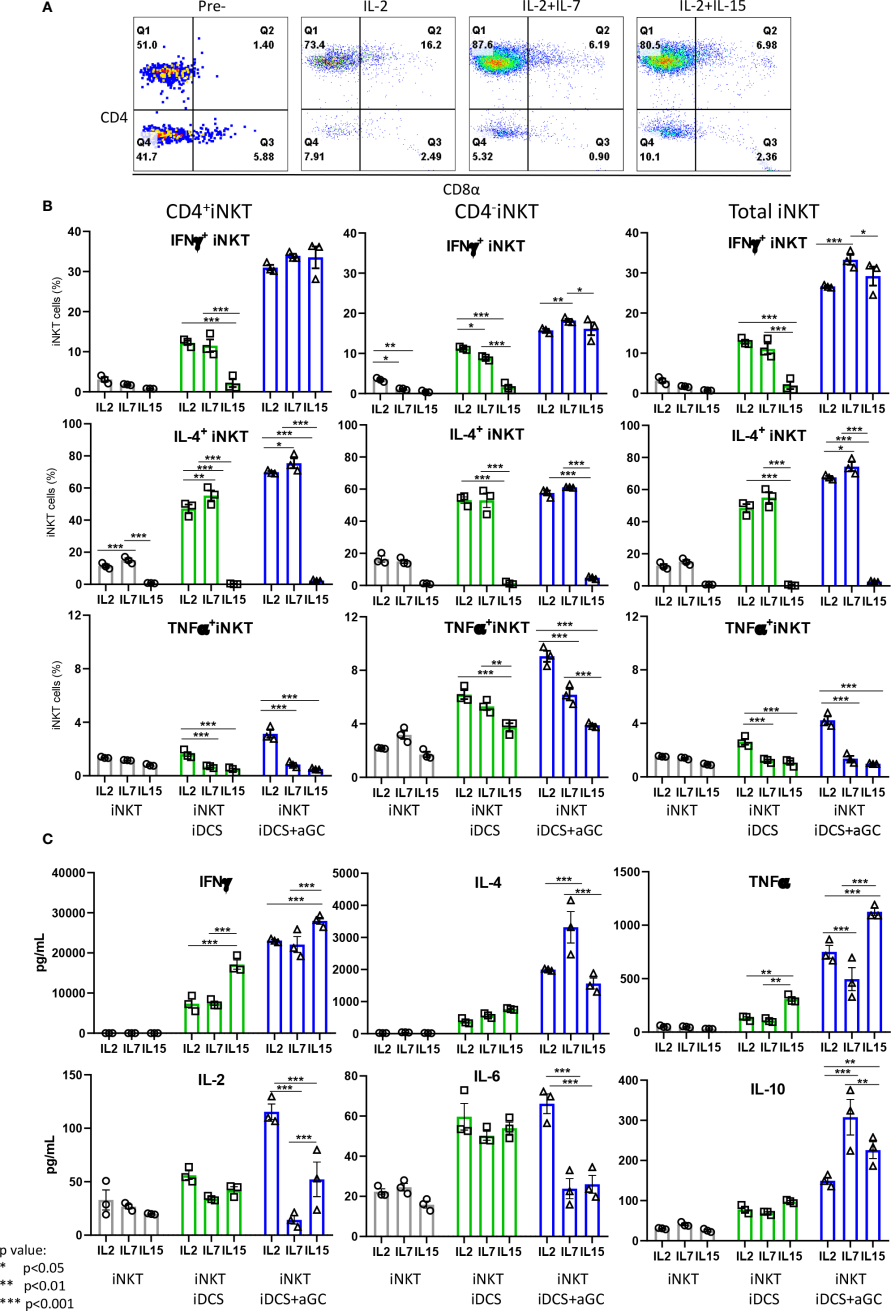
IL-7 during antigenic stimulation enhances Th-2 cytokine expression by expanded human iNKT cells. **(A)** Representative flow cytometry dot plot of iNKT cells expanded in IL-2, IL-2+ IL-7, and IL-2+IL15. The iNKT cells were stimulated by irradiated dendritic cells in the presence or absence of αGalCer for 48 h. For intracellular cytokine production, iNKT cells were incubated with protein transport inhibitor for another 6 h prior to intracellular cytokine staining. The percentages of cytokine ^+^ iNKT cells from the total iNKT cells or CD4^+/-^ iNKT cells were shown **(B)**. The cytokines present in culture supernatant was assessed by a human Th1/Th2/Th17 Cytometric Bead Array kit **(C)**. Experiment was performed with 3 independent donors in triplicates per condition, and representative experiment was presented. Student < test was used to compare the differences of cytokine production between conditions, and represent by *p < 0.05, **p < 0.01 and ***p < 0.001, respectively, not significant are not indicated.

Next, we investigated whether iNKT cells expanded in the presence of IL-2, IL-2+IL7, and IL-2+IL-15 displayed differential cytokine production profile upon TCR-mediated activation – activation considered in physiologic condition (**Figure 8**). As shown in **Figure 8A**, iNKT cells expanded in IL-2+IL-7 contained a higher fraction of CD4^+^ iNKT cells compared to iNKT cells expanded in IL-2 only or IL-2+IL-15. The iNKT cells were co-cultured with irradiated dendritic cells in the presence or absence of αGalCer for 48 h, and cytokine production profile was assessed by intracellular cytokine staining of iNKT cells and Th1/Th2/Th17 cytokine bead array for the presence of secreted cytokine in the culture supernatants (**Figures 8B, C**). First, IFN*γ* ^+^ iNKT cells within CD4^+^ iNKT cells were similar among iNKT cells expanded in three conditions but IL-4^+^ iNKT cells were significantly higher in CD4^+^ iNKT subsets expanded in IL2+IL-7 while they were lower in all subsets of iNKT cells expanded in IL-2+IL-15. The TNFα^+^ iNKT cells decreased in all subsets of iNKT cells expanded in additional IL-7 or IL-15. Next, we investigated cytokines secreted into the culture medium by activated iNKT cells as this assay will provide another insight on the function of iNKT cells. We observe that iNKT cells expanded in IL-2 + IL-7 produced significantly higher level of IL-4 and IL-10 compared to iNKT cells expanded in IL-2 only or IL-2 +IL-15, while iNKT cells expanded in IL-2 plus IL-15 secreted significantly higher level of IFN*γ* and TNFα. Our results suggests that additional IL-7 during antigenic expansion does influence Th1/Th2 cytokine production profile of expanded iNKT cells towards Th2-biased pattern.

In summary, our results suggest that IL-7, but not IL-15, may promote a biased production of Th-2 type cytokines by expanded CD4^+^ iNKT cells compared to CD4^-^ iNKT cells when added to IL-2 during antigenic stimulation.

## Discussion

The iNKT cells are innate-like T lymphocytes that may play an important immunoregulatory role in various infectious and autoimmune diseases as well as cancer as the loss or dysfunction of these cells has been associated with several autoimmune processes (34, 35), cancer (36), and viral infections (37, 38). However, the mechanism of immune-regulation is rather complex than simple. First, iNKT cells can produce an abundant amount of Th-1 type cytokines such as IFNγ and TNFα, which can promote an inflamed immune-microenvironment beneficial for anti-microbial or anti-tumor immunity (39, 40). In addition, these cells can display direct cytolytic activity against infected cells or tumors *via* NK-like effector function (41, 42). Lastly, iNKT cells can trans-activate NK cells to augment additional anti-microbial and anti-tumor immunity (43) and facilitate the cross-priming of T cells through a feedback loop with dendritic cells (44, 45). On the contrast, the immunosuppressive functions of iNKT cells are thought to arise from their ability to produce Th-2 type cytokines (8, 46) and more recently from creating feedback loops involving other immunosuppressive cells like regulatory T cells (Tregs) or myeloid-derived suppressor cells (MDSCs) (21, 47). This plasticity of iNKT cell function, along with extreme paucity of iNKT cells, may pose a challenge when developing iNKT cell-based immunotherapies for the extreme spectrum of diseases where iNKT cell therapy can be justified to promote either inflammation or immune-suppression.

Here, we evaluated three different strategies to expand iNKT cells from peripheral blood – direct stimulation of iNKT cells in PBMCs, stimulation of enriched iNKT cells by irradiated antigen presenting cells, and demonstrated that one-step enrichment of iNKT cells prior to antigenic stimulation was a key step to reliably obtain highly pure iNKT cells after a single round of expansion (**Figures 1** and **2**). Moreover, we found that the choice of antigen presenting cells such as monocyte-derived allogeneic dendritic cells or monocytes-rich autologous PBMCs influenced the expansion of iNKT subsets – e.g., CD4^+^ iNKT cells vs CD4^-^CD8α^-^ iNKT cells, respectively. This is likely because CD4^+^ iNKT cells may depend more on efficient antigenic stimulation than CD4^-^ iNKT cells, further investigation is in need to elucidate a unique requirement for activation of iNKT subsets.

The functional polarization of activated iNKT cells towards either Th-1 or Th-2 type responses can be achieved through several mechanisms. First, the quality of TCR-mediated activation can influence biasing towards Th-1 or Th-2 type responses (48). While strong agonist glycolipid antigens like αGalCer or other Th-1 polarizing αGalCer derivatives can lead to effective anti-microbial/anti-tumor activity from activated iNKT cells, weaker agonists like endogenous glycolipids or Th-2 biasing αGalCer analogs can polarize iNKT cell responses towards Th-2. This biasing mechanism is thought to account for iNKT-cell mediated immune-suppression in several autoimmune processes (49, 50). Besides, several cytokines can influence the outcome of activated iNKT cells (51–53). For example, the presence of IL-12 can drive activated iNKT cells to increase production of IFNγ, and a combination of IL-6, TGFβ, and IL-1β can induce iNKT cells to produce IL-17, a strong inflammatory cytokine (54–56). However, soluble factors that can polarize iNKT cells towards Th-2 type responses are not well known to date.

IL-7 and IL-15 cytokines contribute to the differentiation and maintenance of conventional memory T cell populations (22, 23, 57), and IL-15 has been used to increase the persistency of T cells engineered to express chimeric antigen receptors (53). These cytokines are also critical in the development and homeostasis of murine and human iNKT cells (25, 26). More specifically, thymic development of murine iNKT cells is severely affected in IL-7 knock-out mice, and IL-15 contributes to thymic independent maturation of murine iNKT cells (26, 58, 59). In humans, IL-7 and IL-15 differently affect the homeostasis of CD4^+^ vs CD4^-^ T cells (17, 60). CD4^+^ iNKT cells, from adult and cord blood, constitutively express higher levels of IL-7α receptors and thus proliferate in response to IL-7, whereas IL-15 can better support the proliferation of CD4^-^ iNKT cells (61). More recently, the expression of IL-7α receptor endodomain on CAR T cells have shown to confer better expansion and persistence of IL-7Rα^+^ CAR T cells (53, 62), supporting role of IL-7 in homeostasis of T cells and iNKT cells. (63)

Further, we evaluated if IL-7 and IL-15 can facilitate the selective expansion of CD4^+^ or CD4^-^ iNKT cells respectively and modulate Th-1 or Th-2 type cytokine production. A single antigenic stimulation with αGalCer pulsed dendritic cells and IL-2 only resulted in a robust expansion of CD4^+^ iNKT cells by an average increase of 30.5% (± 17.23%, *p*<0.0001, N=18 donors, hereafter) from the fresh isolated iNKT cells. The incorporation of IL-7 but not IL-15 during *ex vivo* expansion of iNKT cells further facilitated the selective growth of Th-2-biased CD4^+^ iNKT cells in single donor cell product by an average increase of 6.67% (± 7.23%, *p*=0.0001) compared to co-culture condition with IL-2 only. Although this additional increment appears minor but it was consistent as we observed an increase in final iNKT cell products from 16 out of 18 consecutive donors with pairing correlative efficiency of 0.9544 (*p*<0.0001). Given that CD4^+^ iNKT cells expanded by our approach – single antigenic stimulation with IL-2 expressed both IL-7α and IL-15α receptor compared to CD4^-^ iNKT cells (**Supplementary Figure 2**), our results suggest that IL-7α receptor signaling pathway may lead to the transcription of IL-2 common receptor gamma-dependent cytokines including IL-4 through STAT5 [63{Shum, 2017 #32]. In addition*, in silico* analysis confirmed that interconnected signaling pathway among IL7R-STAT5-IL2RG in CD4 T cells (**Supplementary Figure 3**).

In addition, our approach in stimulation with αGalCer pulsed dendritic cells supports a substantial increase in the fraction of CD62L^+^ central memory (CD45RA^-^CD62L^+^) iNKT cells in final cell products (12). This CD62L^+^ iNKT cells are of particular interests as they may persist better than CD62L^-^ iNKT cells *in vivo* when adoptively transferred to immunocompromised mice. Here, we confirmed that dendritic cell mediated stimulation led to a robust expansion of CD62L^+^ CM iNKT cells, especially in CD4^+^ more than CD4^-^ compartment by average 33.6% (± 13.7%, *p*<0.0011) vs 13.6% (± 10.7%, *p*<0.0011), respectively. Interestingly, the addition of IL-15 to the co-culture significantly abrogated this selective expansion of CD62L^+^ iNKT cells in CD4^+^ subset of iNKT cells while the additive IL-7 during stimulation maintained an increased fraction of CD4^+^ iNKT cells with a central memory phenotype (CD45RA^-^CD62L^+^) when compared to iNKT cells expanded with IL-2 alone. These data suggest that CD4^+^ iNKT cells expanded with IL-2 and IL-7 may persist better when adoptively transferred than those expanded with IL-2 and IL-15 (53, 63).

Currently, multiple immunotherapeutic strategies are being utilized to enhance anti-tumor immunity *via in vivo* expansion of iNKT cells through the delivery of agonist glycolipids or antigen presenting cells loaded with αGalCer (19). Another approach is to perform adoptive transfer of *ex vivo* expanded iNKT cells with or without genetic modifications (19, 53). These approaches have primarily captivated Th-1 type responses from iNKT cells and may not be ideal to treat various autoimmune diseases. Interestingly, our approach to prepare iNKT cells may optimize Th-2 polarization of final iNKT cell products by increasing fraction of Th-2 polarized CD4^+^ subset of iNKT cells compared to other approaches such as direct antigenic stimulation of iNKT cells from PBMCs using αGalCer and IL-2, or CD3/CD28 mediated stimulation of enriched iNKT cells (13, 64). Likewise, additional IL-7 during dendritic cell mediated antigenic stimulation may further aid Th-2 biased cytokine production of iNKT cell products by potentiating the selective expansion of CD4^+^ iNKT cells. More importantly, cytokine production profile of expanded iNKT cell products showed that IL-2 in combination with IL-7 consistently and significantly increased the fraction of IL-4^+^ or IL-13^+^ iNKT cells by 5.8% (± 6.8%, *p*=0.0011) or 10.4% (± 12.3%, *p*=0.0003) within CD4^+^ subsets from iNKT cell products when compared to those cultured using IL-2 only. These expanded iNKT cells with IL-2/IL-7 maintained Th-2 biased cytokine production profile upon antigen specific stimulation. As a result of selective expansion of Th-2 biased CD4^+^ iNKT cells, we anticipate the iNKT cell products prepared from a single antigenic stimulation with dendritic cells in the presence of IL-2 and IL-7 display Th-2 biased cytokine production profile.

In conclusion, we demonstrated that one-step enrichment of iNKT cells prior to antigenic stimulation was a key element to obtain highly pure iNKT cells after ex vivo expansion, and the incorporation of IL-7 during dendritic cell mediated, antigenic stimulation can significantly expand Th-2 biased, CD62L^+^CD4^+^ iNKT cells in *ex vivo* without abrogating the purity and quantity of iNKT cells. These *ex vivo* expanded, highly enriched Th-2^+^CD62L^+^CD4^+^iNKT cells may have a tremendous potential as novel cell therapeutics for several autoimmune diseases where quantitative and qualitative defects of iNKT cells are linked to the pathogenesis of the disease (65–67).

## Data Availability Statement

The raw data supporting the conclusions of this article will be made available by the authors, without undue reservation.

## Ethics Statement

The studies involving human participants were reviewed and approved by the University of Texas M.D. Anderson Institutional Review Committee. The patients/participants provided their written informed consent to participate in this study.

## Author Contributions 

AT-O, H-WC, W-RV, and MC performed experiments. AT-O, H-WC, MC, and JI interpreted results. AT-O, JI, H-WC, SP, S-EL, and MC wrote manuscripts. All authors contributed to the article and approved the submitted version.

## Funding

This work was supported by T32-CA009666 (JI), M.D. Anderson Advanced Scholar (JI), New Investigator Award from American Society of Blood and Marrow Transplantation (JI), Amy Strelzer Manasavit Research Scholar from National Marrow Donor Program (JI), MD Anderson Institutional Start-Up (JI), and Cancer Prevention and Research Institute of Texas, RP200023, to JI. The South Campus Flow Cytometry & Cell Sorting Core was supported by NCI P30CA016672.

## Conflict of Interest

The authors declare that the research was conducted in the absence of any commercial or financial relationships that could be construed as a potential conflict of interest.

## Supplementary Material

Supplementary Figure 1 Viability of expanded iNKT cells. Viability of expanded iNKT cells co-cultured with allogeneic DCs in presence of αGalCer and IL-2, IL-2+IL-7, or IL-2+IL-15 were measure by dye exclusion. The percentage of viable cells in culture was determine by amine-reactive membrane impermeable dye used to discriminate viable from non-viable iNKT cells based on fluorescence intensity (BD Horizon Fixable Viability stain 620). Fixable Viability stain was add while the surface antibodies cocktail, then the percentage of viability of expanded iNKT cells were determined from total lymphocyte CD3^+^ population.

Supplementary Figure 2 IL7R (CD127) and IL15R (CD215) expression in expanded iNKT cells. **(A)** Immunophenotypic (CD4 vs CD124) analysis of expanded iNKT cells (αGalCer+IL-2) are shown. **(B)** Immunophenotypic (CD4 vs CD215) analysis of expanded iNKT cells (αGalCer+IL-2) are shown. Horizontal orange lines represent mean values; *p* values is shown. Single symbol represents a value from a single donor.

Supplementary Figure 3 IL7R network. To identify proteins interacting with IL7R, we searched fort known and predicted protein-protein interaction using the STRING protein-protein-interaction database (https://string-db.org/). This approach result in an IL7R network that consist of IL7R and proteins interactions: JAK3, IL7, STAT5A, STAT5B, STAT3, JAK1, IL2RG, TSLP, CRLF2 and CD4.
