# Corrigendum: IL-7 During Antigenic Stimulation Using Allogeneic Dendritic Cells Promotes Expansion of CD45RA^-^CD62L^+^CD4^+^ Invariant NKT Cells With Th-2 Biased Cytokine Production Profile

**DOI:** 10.3389/fimmu.2021.689959

**Published:** 2021-04-20

**Authors:** Abel Trujillo-Ocampo, Hyun-Woo Cho, Sumedha Pareek, Wilfredo Ruiz-Vazquez, Michael Clowers, Sung-Eun Lee, Jin S. Im

**Affiliations:** ^1^ Department of Stem Cell Transplantation and Cellular Therapy, University of Texas MD Anderson Cancer Center, Houston, TX, United States; ^2^ Department of Hematology, Seoul St. Mary’s Hospital, College of Medicine, The Catholic University of Korea, Seoul, South Korea

**Keywords:** *ex vivo* expansion, human iNKT cells, Th2 polarization of expanded iNKT cells, IL-2, IL-7, IL-15, αGalCer, CD62L^+^ iNKT cells

In the original article, we neglected to include the funder Cancer Prevention & Research Institute of Texas, RP200023, to JI.

The authors apologize for this error and state that this does not change the scientific conclusions of the article in any way. The original article has been updated.

