# Supplementary figures and images for: IL-7 During Antigenic Stimulation Using Allogeneic Dendritic Cells Promotes Expansion of CD45RA-CD62L+CD4+ Invariant NKT Cells With Th-2 Biased Cytokine Production Profile
Source: Front Immunol. 2020 Nov 27;11:567406. doi: 10.3389/fimmu.2020.567406 (PMC7728799; doi:10.3389/fimmu.2020.567406)

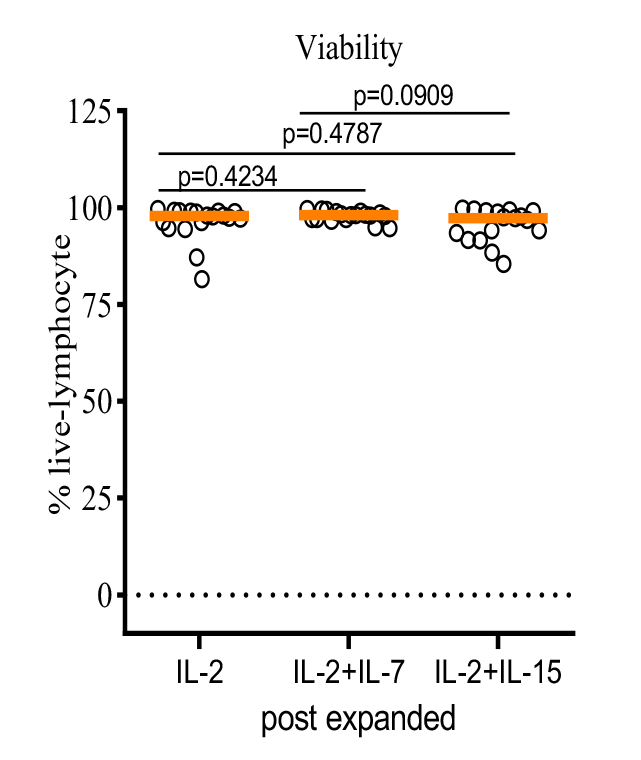

Supplement: Supplementary Figure 1 — Viability of expanded iNKT cells. Viability of expanded iNKT cells co-cultured with allogeneic DCs in presence of αGalCer and IL-2, IL-2+IL-7, or IL-2+IL-15 were measure by dye exclusion. The percentage of viable cells in culture was determine by amine-reactive membrane impermeable dye used to discriminate viable from non-viable iNKT cells based on fluorescence intensity (BD Horizon Fixable Viability stain 620). Fixable Viability stain was add while the surface antibodies cocktail, then the percentage of viability of expanded iNKT cells were determined from total lymphocyte CD3+ population. [file Image_1.tif]

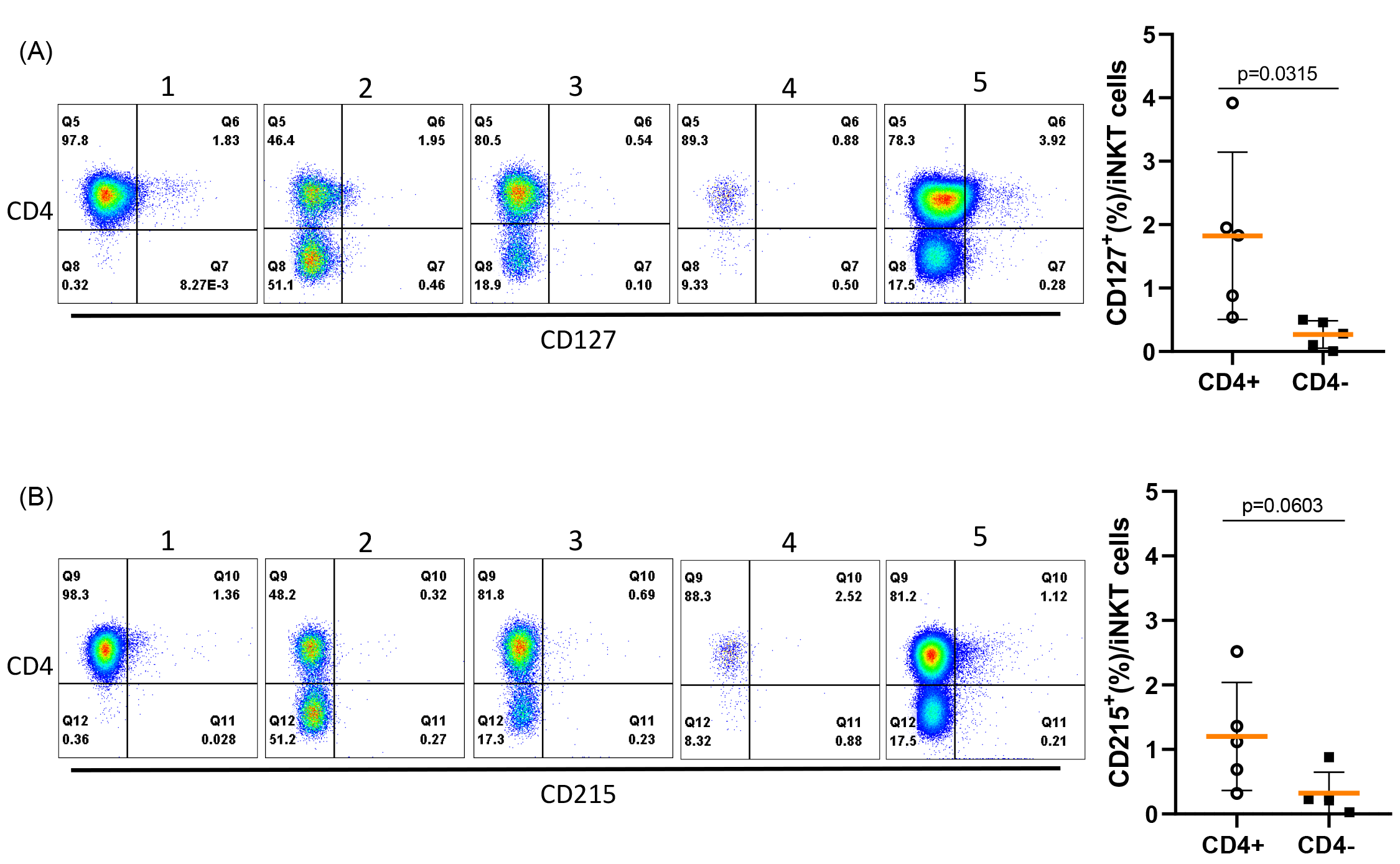

Supplement: Supplementary Figure 2 — IL7R (CD127) and IL15R (CD215) expression in expanded iNKT cells. (A) Immunophenotypic (CD4 vs CD124) analysis of expanded iNKT cells (αGalCer+IL-2) are shown. (B) Immunophenotypic (CD4 vs CD215) analysis of expanded iNKT cells (αGalCer+IL-2) are shown. Horizontal orange lines represent mean values; p values is shown. Single symbol represents a value from a single donor. [file Image_2.tif]

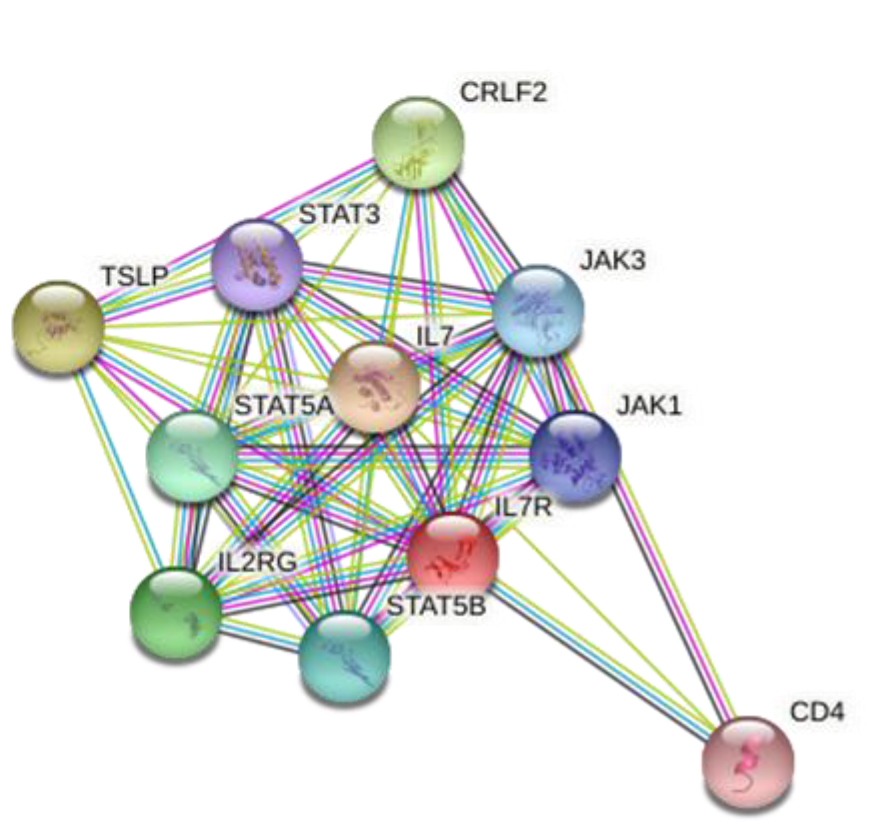

Supplement: Supplementary Figure 3 — IL7R network. To identify proteins interacting with IL7R, we searched fort known and predicted protein-protein interaction using the STRING protein-protein-interaction database (https://string-db.org/). This approach result in an IL7R network that consist of IL7R and proteins interactions: JAK3, IL7, STAT5A, STAT5B, STAT3, JAK1, IL2RG, TSLP, CRLF2 and CD4. [file Image_3.tif]
